# Monocytes from inflammatory arthritis patients accumulate methotrexate and their transcriptome predicts methotrexate clinical response

**DOI:** 10.1007/s00018-026-06162-9

**Published:** 2026-03-17

**Authors:** Israel Ríos, María Teresa Schiaffino, Baltasar López-Navarro, Ana Triguero-Martínez, Marry Lin, Mónica Torres-Torresano, Eduard A. Struys, Susana Álvarez, Manuel Román, Francisco Abad-Santos, Santos Castañeda, Noelia Garcia-Castañeda, Robert de Jonge, Gerrit Jansen, Isabel Castrejón, Isidoro González-Álvaro, Amaya Puig-Kröger

**Affiliations:** 1 Immunometabolism and Inflammation Laboratory, Hospital General Universitario Gregorio Marañón, Instituto de Investigación Sanitaria Gregorio Marañón, Madrid, Spain; 2 Myeloid Cell Laboratory, Centro de Investigaciones Biológicas, CSIC, Madrid, Spain; 3 Rheumatology Service, Hospital Universitario de La Princesa, IIS Princesa, Madrid, Spain; 4 Laboratory of Specialized Research and Diagnostics, Amsterdam University Medical Center, Amsterdam, The Netherlands; 5 Molecular Biology Laboratory, Hospital General Universitario Gregorio Marañón, Instituto de Investigación Sanitaria Gregorio Marañón, Madrid, Spain; 6 School of Experimental Sciences, Biosciences Research Institute, Universidad Francisco de Vitoria, Madrid, Spain; 7 Clinical Pharmacology Department, Hospital Universitario de La Princesa, IIS Princesa, Madrid, Spain; 8 Pharmacology Department, School of Medicine, Universidad Autónoma de Madrid, Madrid, Spain; 9 Centro de Investigación Biomédica en Red de Enfermedades Hepáticas y Digestivas (CIBERehd), Instituto de Salud Carlos III, Madrid, Spain; 10 Department of Rheumatology and Clinical Immunology, Amsterdam Rheumatology and Immunology Center, Amsterdam University Medical Center, Amsterdam, The Netherlands; 11 Rheumatology Service, Hospital General Universitario Gregorio Marañón, Instituto de Investigación Sanitaria Gregorio Marañón, Madrid, Spain; 12 Medicine Department, School of Medicine, Universidad Complutense de Madrid, Madrid, Spain

**Keywords:** Inflammatory arthritis, Methotrexate, Monocytes, Aryl hydrocarbon receptor

## Abstract

Monocytes and monocyte-derived macrophages play a key pathogenic role in inflammatory arthritis. Methotrexate (MTX) is the first-line disease-modifying antirheumatic drug (DMARD) for arthritis, yet the mechanisms and kinetics of its effects on monocyte/macrophages remain poorly understood. We have now investigated the temporal dynamics of the MTX's anti-inflammatory action by initially performing a phase I clinical trial (METOMAC) on healthy individuals following a single MTX dose, which revealed that MTX plasma levels peak at 1 h after MTX treatment, with maximal suppression of LPS-induced IL-1β and IL-6 in whole blood 5 days after MTX exposure. Building on these findings, we performed an observational clinical study (METOMAC-PAC) on 29 DMARD-naïve early arthritis patients receiving a weekly dose of MTX over a 3 month period. The METOMAC-PAC study revealed that MTX polyglutamates (MTX-PG) accumulates in peripheral blood monocytes, where MTX-PG is detected as early as 5 days post-treatment, and modulates the monocyte gene profile after 4 MTX doses, with specific enrichment of the aryl-hydrocarbon receptor (AhR)-molecular signature and genes coding for anti-inflammatory factors. Importantly, stratification of METOMAC-PAC patients according to their clinical response revealed that Good Responders exhibit increased expression of “non-classical monocyte”-specific genes (*MAF*, *FCGR3B*, *ICAM4*) both before and after MTX treatment, while Partial Responders patients showed a higher baseline expression of genes preferentially expressed by “classical monocytes”. Our results elucidate the kinetics of the anti-inflammatory action of MTX, demonstrate that MTX modulates the monocyte transcriptional signature *in vivo*, and identify “non-classical monocyte”-associated genes as predictors for an effective MTX clinical response.

**Graphical abstract:**

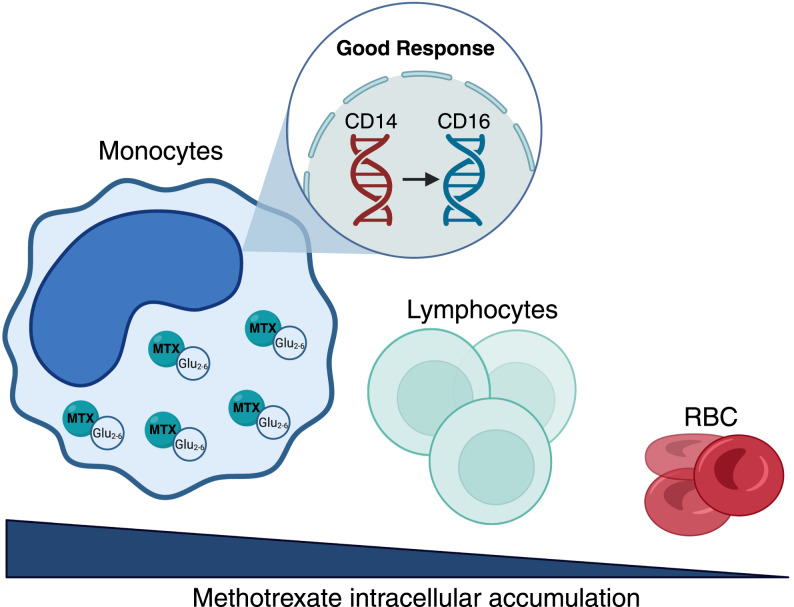

**Supplementary Information:**

The online version contains supplementary material available at 10.1007/s00018-026-06162-9.

## Introduction

Methotrexate (MTX) monotherapy, as well as its combination with other synthetic or biologic disease-modifying anti-rheumatic drugs (DMARDs), continues to be the anchor drug for patients with rheumatoid arthritis (RA) and psoriatic arthritis (PsA), with proved efficacy and safety, low relative costs and the possibility of individualized dosing [1–4]. Following low-dose administration, MTX typically reaches peak plasma concentrations after 1–2 h, and almost completely disappears from the circulation by 24 h [5]. Cellular uptake of MTX in immune-related cells is mediated by several folate transporters including the reduced folate carrier (RFC, *SLC19A1*), proton coupled folate transporter (PCFT, *SLC46A1*) and folate receptor beta (FRβ, *FOLR2*) [6–9]. Once internalized, MTX is converted into MTX-polyglutamates (MTX-PG), a process that is catalyzed by the enzyme folylpolyglutamate synthetase (FPGS), which sequentially adds glutamic acid residues to MTX, and is one of the critical factors for the therapeutic action of MTX [10]. In fact, MTX-PG exhibits enhanced intracellular retention and displays enhanced pharmacological efficacy and more potent inhibition of its target enzymes than non-polyglutamylated MTX [6]. Red blood cells (RBCs) MTX-PG can be used for therapeutic drug monitoring and a meta-analysis showed that particularly MTX-PG3 accumulation correlated with good clinical response [11, 12]. Recently, it has been determined that peripheral blood mononuclerar cells (PBMCs) accumulated tenfold to 30-fold higher levels of total MTX-PGs per cell than RBCs over 6 months of MTX therapy in RA patients [12, 13]. However, whether MTX-PG concentration in PBMCs or PBMC subpopulations correlates with therapeutic response is unknown, even though these cells are involved in the pathophysiology of immune-mediated inflammatory diseases.

Monocytes and monocyte-derived macrophages play a key pathogenic role in inflammatory arthritis [14–16]. Inflammatory monocytes that infiltrate the synovium differentiate into macrophages along RA evolution [16]. Despite its widespread use, the mechanism of MTX's anti-inflammatory action in innate immune cells is not fully understood. Our group has demonstrated that response to MTX depends on the polarization state of monocyte-derived macrophages [17], in which MTX establishes a state of tolerance against pro-inflammatory stimuli *in vitro* [18]. Thus, MTX-treated macrophages exhibit an A20-dependent decreased production of proinflammatory cytokines when exposed to TNFα or synovial fluid from RA patients. In agreement with its ability to limit macrophage responses to danger signals *in vitro*, MTX is capable of inducing a state of tolerance in mice *in vivo* [18]. To assess the immunological effects of MTX treatment in monocytes from patients with inflammatory arthritis *in vivo*, we have evaluated MTX-PG accumulation dynamics upon MTX treatment, as well as the influence of MTX-PG levels on the molecular inflammatory state of monocytes *in vivo*. Our results show that MTX-PG accumulates in monocytes within five days after the start of MTX treatment, and demonstrate that MTX modulates the monocyte transcriptional signature at later timepoints by specifically affecting the AhR-dependent gene profile. Besides, analysis of the correlation between the MTX transcriptional effects and MTX clinical efficacy has allowed the identification of “non-classical monocyte”-associated genes as predictors for effective MTX clinical response, thus highlighting the potential role of “non-classical monocytes” in mediating the MTX response.

## Materials and methods

### Pilot study

Six healthy volunteers (100% men; aged from 21 to 34) were enrolled and followed-up between April to June 2018 in the open phase I clinical trial (CT) METOMAC (EUDRA-CT: 2017–002902-11). This CT was approved by the Ethical Committee of Hospital Universitario La Princesa (February 8th, 2018) and the Spanish Agency of Medicines and Healthcare (AEMPS, March 14th, 2018). METOMAC was carried out under close medical supervision at the Clinical Trials Unit (CTU) of the Institute for Health Research La Princesa. Given that MTX may have abortive and/or teratogenic effects [19], the CTU staff recommended the study be conducted exclusively in healthy male volunteers. Laboratory tests were performed at baseline and at the end of the study, and no relevant adverse events were detected. Heparinized blood was drawn before administration of MTX and 3 h, 24 h and 120 h (5 days) after single oral dose (20 mg) of MTX (Wyeth Farma, Spain). Peripheral blood was maintained for 15 h at room temperature and treated with PBS or LPS (10 ng/ml, 0111:B4 strain, InvivoGen), plasma was recovered using standard procedures and evaluated for the presence of IL-6 and IL1β (Biolegend, Germany). For pharmacokinetic, MTX plasma concentrations were determined before (t = 0), and after 1 h, 2 h, 3 h, 4 h, 6 h, 24 h and 120 h MTX administration at the Pharmacology Service of Hospital Universitario de La Paz (Madrid, Spain).

### Observational clinical study

29 DMARD-naïve patients (69% female) with inflammatory arthritis (75% RA and 25% peripheral polyarticular PsA) starting MTX therapy were enrolled from November 2019 until June 2024 in Hospital Universitario La Princesa and Hospital General Universitatrio Gregorio Marañón, Madrid, Spain. The study was conducted in accordance with the Declaration of Helsinki, was approved by the AEMPS (August 05th 2019) and the Ethical Committee of Hospital Universitario La Princesa and Hospital General Universitario Gregorio Marañón (protocol code: METOMAC-PAC, IGA-MET-2019–01). Starting MTX dose was 12.5 mg/week (median) and folic acid was prescribed at 5 mg/week (24 h after MTX). 30 ml of peripheral blood was extracted in lithium heparinized tubes before the start of MTX treatment (pre-MTX), 5 days after the first MTX intake (1 × MTX, in accordance to results from METOMAC pilot study) and 5 days after the fourth MTX dose (4 × MTX). Patients were evaluated by a rheumatologist at baseline and at month 1 and 3 after initiation of MTX. Clinical characteristics of patients were collected including age, sex, rheumatoid factor (RF) and anti-citrullinated protein antibodies (ACPA) status, diet habits that may impact MTX effect as coffee consumption and disease activity measures (disease activity score on 28 joint count through erythrocyte sedimentation rate [DAS28-ESR]) at baseline and at the 3-month follow up. Patients were classified as good or partial responders according to the clinical evaluation by the treating rheumatologist after 3 months of MTX therapy: all patients improved their DAS28-ESR at 3-months visit, so we defined good responders (GR) patients those who showed adequate control of disease activity and no need to increase MTX, while partial responders (PR) exhibited active disease and required increasing the MTX dose.

### Cell isolation

PBMCs were isolated from whole blood over a Lymphoprep (Nycomed Pharma) gradient. Monocytes (CD14^pos^) were purified from PBMC by magnetic cell sorting using CD14 microbeads (Miltenyi Biotech), cells were manually counted with Trypan blue and RNA (1 × 10^6^ cells) was isolated with RNeasy Micro Kit (QIAGEN) and pellets (2 × 10^6^ cells) were snap-frozen and stored at − 80 °C. Pellets of the CD14 negative fraction (CD14^neg^, mainly lymphocytes) were also isolated. Packed erythrocytes were collected from EDTA whole blood tubes by centrifugation at 1,700 xg for 10 min at room temperature and stored at − 80 °C until analysis.

### Chemicals

Routine chemicals were obtained with the highest grade of purity from Sigma Aldrich (Zwijndrecht, The Netherlands or St. Louis, MO, USA). Methotrexate (MTX-PG_1_, Emthexate PF, 100 mg/mL) was obtained from Teva Pharmachemie (Haarlem, The Netherlands). ^15^N-L-glutamic acid (98% ^15^N) was obtained from Sigma-Aldrich (Zwijndrecht, The Netherlands). ^13^C_5,_^15^N-labeled MTX-PG_1-5_ stable isotope-labeled internal standards were obtained from Pepscan (Lelystad, The Netherlands).

### Methotrexate polyglutamates (MTX-PG_1-5_) analysis in CD14^pos^ and CD14^neg^ cells

MTX-PG levels were assessed following a slightly modified procedure described by Hebing et al. [13, 20]. Briefly, stable isotope ^13^C_5,_^15^N-labeled MTX-PG_1-5_ internal standards (6 nmol/L; Pepscan, Lelystad, The Netherlands) were added to the cell pellets (2–3 × 10^6^ cells), followed by lysis and deproteination with 40 μl 16% (v/v) perchloric acid on ice. Upon centrifugation (15,000 xg, 15 min, 4 °C), the supernatant was collected and filtered (0.22 µm PVDF, Merck, Germany). The resulting filtrate was subjected to solid-phase extraction, followed by analysis with UPLC-ESI–MS/MS. Results were calculated using calibration curves and reported as fmol MTX-PGn/10^6^ cells. Total MTX-PG concentrations were calculated as the sum of MTX-PG_1-5_.

### MTX-PG_1-5_ analysis in red blood cells (RBCs)

Quantification of individual MTX-PG_1-5_ in packed RBCs was performed as previously described by den Boer et al. [21]. In short, ^13^C_5,_^15^N-labeled MTX-PG_1-5_ internal standards were added to the RBCs, followed by lysis and deproteination with perchloric acid. After filtration, MTX-PG_1-5_, were analyzed by UPLC-ESI–MS/MS. Results are reported as fmol MTX-PGn/10^6^ RBCs. Total MTX-PG concentrations were calculated as the sum of MTX-PG_1-5_.

### FPGS assay

FPGS enzymatic activities of CD14^pos^ and CD14^neg^ cells were analyzed essentially as described by Muller et al. [22]. In short, frozen cell pellets (1.4–7.6 × 10^6^ cells) were resuspended in 250 μl FPGS extraction buffer, followed by sonication (Sonoplus Mini 20) on ice for 2 × 5 s with 30 s intervals at amplitude 90%. After centrifugation at 4 °C in Eppendorf centrifuge (10 min, 14,000 xg), 20–75 μg protein extract was added to the assay mixture containing 250 μmol/L MTX-PG_1_ (Teva Pharmachemie) and 4 mmol/L ^15^N-L-glutamic acid (Sigma-Aldrich). The product of the enzymatic conversion of MTXPG1 i.e. ^15^N -MTX-PG_2_ was measured by UPLC-ESI–MS/MS (TSQ Quantiva, Thermo Scientific) and results for FPGS activities were reported as pmol ^15^N-MTX-PG_2_-formed/h/mg protein. CCRF-CEM and FPGS-deficient CEM/R30dm leukemic cell lines were used as positive and negative controls respectively for FPGS activity.

### RNA-seq and bioinformatic analysis

Total RNA was processed at BGI (https://www.bgitechsolutions.com), where library preparation, fragmentation and sequencing were performed using the BGISEQ-500 platform. An average of 4.75 Gb bases were generated per sample and, after filtering, clean reads were mapped to the reference (UCSC Genome assembly hg38.p13) using Bowtie2. Raw data with adapter sequences or low-quality sequences was filtered with SOAPnuke software filter parameters: "-n 0.01 -l 20 -q 0.4 –adaMR 0.25 –polyX 50 –minReadLen150″. The average mapping ratio with reference genome was 99.16%, the average mapping ratio with gene was 79.65%; 17176 genes were identified. Differential gene expression was assessed by using DEseq2 v1.44.0 algorithms using the parameters Fold change > 1 and *p-*value < 0.05. Principal Component Analysis (PCA) and Partial Least Squares-Discriminant Analysis (PLS-DA) on the RNAseq from monocytes was preprocessed using the vst function for normalization of read counts and then processed using DEseq2 v1.44.0 and mixOmics v6.28.0, respectively. Gene Ontology term enrichment analysis was conducted by using genes with a Fold change > 1 and *p-*value < 0.05 using R package clusterProfiler v4.12.6, only the top 10 enriched pathways were shown. Gene set enrichment analysis (GSEA) [23] was performed using the clusterProfiler v4.12.6 and enrichplot v1.24.4 package. The gene sets available at the website as well as gene sets generated from publicly available transcriptional studies were used and are listed and described in Supplementary Table 2. Data reported in this publication have been deposited in NCBI's Gene Expression Omnibus and are accessible through GEO Series accession numbers GSE279719 and GSE289101.

### Predictive modeling with support vector machines (SVM)

Machine learning approaches, including linear support vector machines (SVM) and random forest (RF) classifier were applied using the caret v7.0–1 package in R. The SVM classifier was trained with the svmLinear method (liblinear backend) after centering and scaling predictor variables, while the RF classifier was built using the randomForest implementation with 500 trees, with the number of predictors considered at each split (mtry) tuned over a predefined grid and optimized according to the area under the ROC curve (AUC). Both models performance were evaluated using leave-one-out cross-validation (LOOCV), whereby each sample was iteratively left out as a test case while the model was trained on the remaining samples. Classification accuracy, sensitivity, and specificity were calculated across LOOCV folds. To assess whether predictive performance exceeded chance, we conducted a label permutation test. The good responder/partial responder labels were randomly permuted 500 times, and the LOOCV classification accuracy was recomputed for each shuffled dataset using the same SVM procedure. The empirical *p-value* was calculated as the proportion of permuted accuracies greater than or equal to the observed accuracy. Variable importance scores for genes and clinical covariates were obtained from both classifiers. For the SVM, weights from the linear separating hyperplane were extracted. For the RF, importance was quantified as the mean decrease in Gini index across trees. The top predictors were visualized using pheatmap v1.0.13 package.

### Statistical analysis

Statistical analysis was done using GraphPad Prism. Most quantitative variables followed a non-normal distribution, so they were represented as median and interquartile range (IQR) and the Mann Whitney or Kruskal–Wallis tests were used to analyse significant differences. *p* value < 0.05 was considered significant (*, *p* < 0.05; **; *p* < 0.01, ***; *p* < 0.001; ****p < 0.0001). To analyse whether there was an association between the response to MTX and MTX-PGs accumulation in monocytes or with gene expression (*FCGR3B*, *ICAM4*, *APOBEC3A*, *CD226* and *MAF*), logistic regression models were performed using the logit command of Stata 14.1. The model was adjusted for sex and MTX dose. Due to the small sample size, only a limited number of covariates were included to avoid overfitting and unstable estimates.

## Results

### Kinetics of the anti-inflammatory action of initial MTX intake: METOMAC trial

**Fig. 1:**
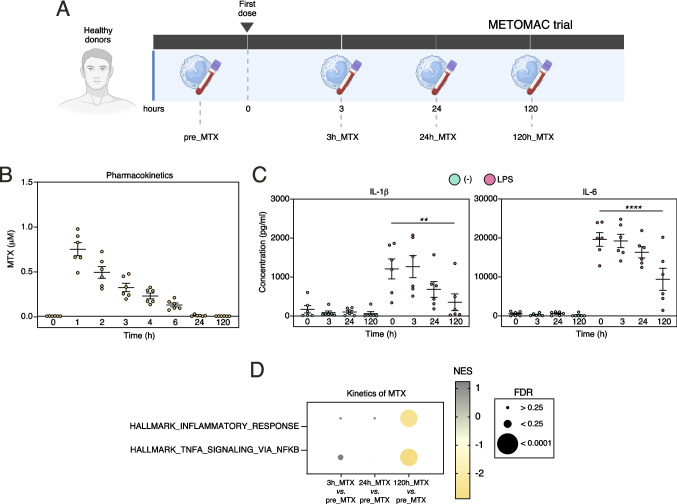
METOMAC trial: Kinetics of the anti-inflammatory action of initial MTX intake. **A** Schematic representation of the experiments performed in METOMAC clinical trial. Blood was analyzed from healthy individuals (*n* = 6) before MTX administration (0 h), and 3 h, 24 h and 120 h after 20 mg MTX intake. **B** MTX pharmacokinetics determined before MTX administration (t = 0), and 1 h, 2 h, 3 h, 4 h, 6 h, 24 h and 120 h after MTX dosage. **C** Cytokine concentration in plasma isolated from blood (0 h, 3 h, 24 h and 120 h after MTX) challenged *ex vivo* with LPS for 15 h (**p < 0.01, ****p < 0.0001, one-way ANOVA with Tukey´s post hoc test). **D** Enrichment of the indicated ranked comparison (3h_MTX vs pre_MTX; 24h_MTX vs pre_MTX and 120h_MTX vs pre_MTX) monocyte transcriptomes using the Hallmarks v7.2 data set available at the GSEA web site. The intensity of color increases with the enrichment of the gene signature. FDRq value is defined by the size of the corresponding circle

Arthritis patients receive a weekly dose of MTX as a first-line DMARD [1]. However, the kinetics of the anti-inflammatory action of MTX after the initial dose is unknown. To address this issue, we designed a phase I clinical trial (METOMAC) in which peripheral blood from 6 healthy men receiving a single dose of MTX was analyzed at distinct time points before (0 h) and after drug intake (3 h, 24 h, 120 h) (Fig. 1A, Suppl. Table 1). Pharmacokinetics parameters indicated that MTX plasma levels peaked 1 h and were undetectable after 24 h, as expected [5, 24] (Fig. 1B). However, and regarding MTX anti-inflammatory effects in peripheral blood, maximal reduction of LPS-induced IL-1β and IL-6 levels in whole blood was observed after 120 h (Fig. 1C). Indeed, and despite the small sample size of the trial, RNA-seq on monocytes isolated at the distinct timepoints revealed a robust negative enrichment of genes associated with the Hallmark GSEA terms “Inflammatory_Response” and “TNF_Signaling_via_NFKB” (Fig. 1D). Therefore, since MTX did not alter the number of circulating lymphocytes, monocytes, neutrophils or platelets at any timepoint (Suppl. Figure 1), METOMAC trial allowed us to conclude that anti-inflammatory effect can be detected 5 days after MTX intake.

### METOMAC-PAC observational study: MTX-PG accumulation in monocytes

**Fig. 2:**
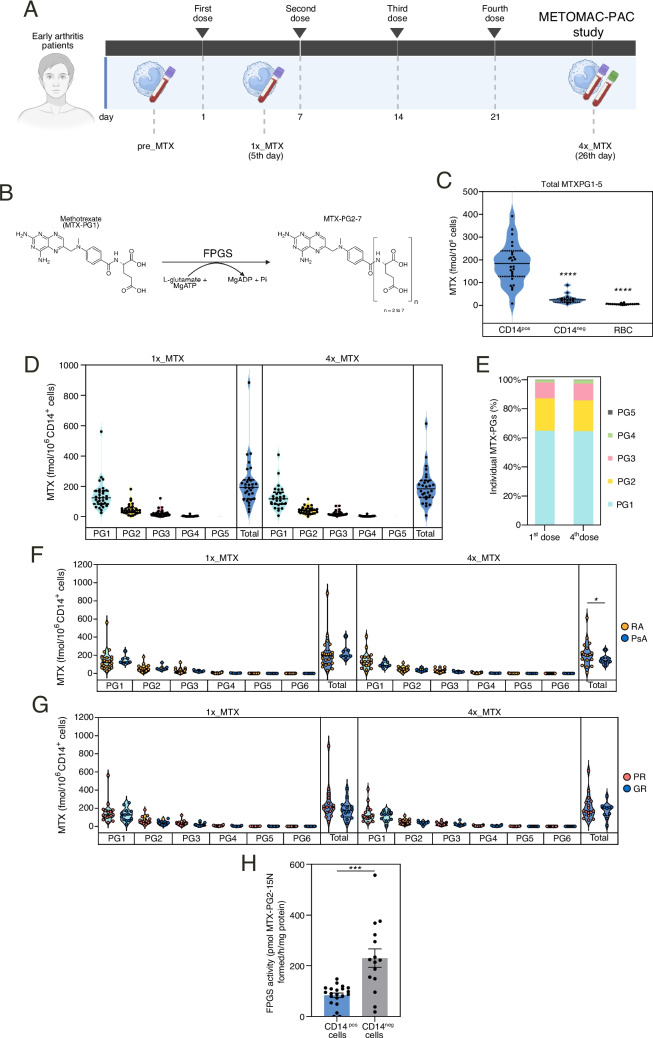
METOMAC-PAC study: MTX-PG in monocytes from early arthritis patients. **A** Schematic representation of the experiments. Monocytes were isolated at baseline (pre_MTX), and following 5 days (1x_MTX, one-dose) and 26 days (4x_MTX, four-doses) of low-dose MTX treatment. MTX-PG concentrations and RNAseq was determined. **B** Scheme depicting MTX polyglutamylation. Folylpolyglutamate synthetase (FPGS) catalyzes MTX by conjugating glutamate moieties to MTX monoglutamate (MTX-PG1) resulting in MTX-polyglutamates (MTX-PG2-7). **C** Total MTX-PG in CD14^pos^ cells (monocytes, *n* = 26), CD14^neg^ cells (*n* = 14) and RBC (*n* = 21) in 4x_MTX time point (****p < 0.0001, one-way ANOVA with Tukey´s post hoc test). **D-E** Individual MTX-PG concentrations in monocytes in 1x_MTX (*n* = 29) and 4x_MTX (*n* = 26) time points. **F** MTX-PG concentrations in CD14^pos^ monocytes from rheumatoid arthritis (RA, orange, *n* = 22) and psoriatic arthritis (PsA, blue, *n* = 7) patients in 1x_MTX and 4x_MTX time points (*p* < 0.05, one-way ANOVA with Fisher´s post hoc test). **G** MTX-PG concentrations in monocytes (CD14^pos^) from MTX-partial responders (PR, pink, *n* = 16) and good responders (GR, blue, *n* = 13) patients in 1x_MTX and 4x_MTX time points (*p* < 0.05, one-way ANOVA with Fisher´s post hoc test). For C-F, PG1: MTX-PG1; PG2:MTX-PG2; PG3: MTX-PG3; PG4: MTX-PG4; PG5: MTX-PG5; PG6: MTX-PG6 **H** FPGS activity in CD14^pos^ cells (monocytes, *n* = 21), and CD14^neg^ cells (*n* = 15) at baseline (pre_MTX) (***p < 0.001, paired t-test)

**Fig. 3:**
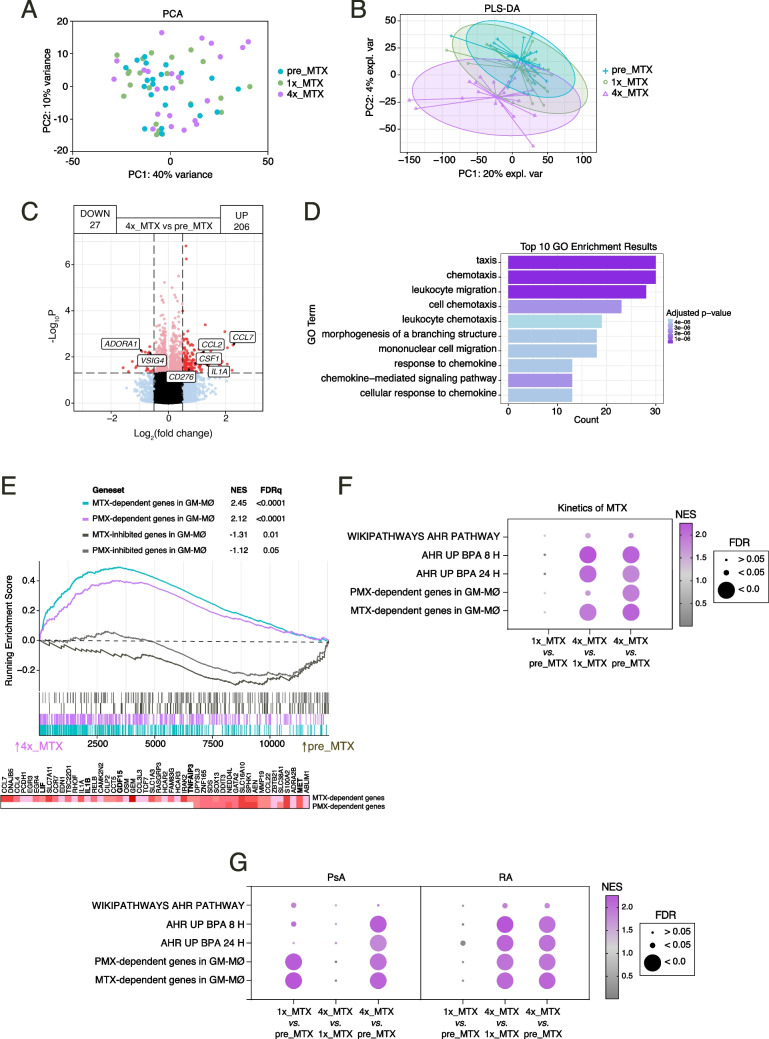
METOMAC-PAC study: Longitudinal effect of MTX in the transcriptome of monocytes from arthritis patients. Principal Component Analysis (PCA) (**A**) and Partial Least Squares-discriminant Analysis (PLS-DA) (**B**) analysis of monocyte transcriptome from 22 patients (only RNA from patients with complete visits) at the three timepoints (pre_MTX, 1x_MTX and 4x_MTX). Sample points coloured by sample timepoint. Volcano plot (**C**) and gene ontology (**D**) of RNAseq results showing the MTX-induced gene expression changes (*p* < 0.05) after 26 days of MTX treatment (4x_MTX). **E** GSEA on the ranked comparison of the 4x_MTX versus pre_MTX monocyte transcriptomes, using the genes significantly modulated by MTX (GSE71253) and the anti-folate pemetrexed (PMX) (GSE159349) in GM-CSF-primed macrophages (GM-MØ) as data set. Normalized Enrichment Score (NES) and False Discovery Rate (FDRq) are indicated. Leading edge analysis of the GSEA of the genes that define anti-folate response on the ranked comparison of the transcriptomes of 4x_MTX versus pre_MTX groups is shown in the bottom panel. In the heatmap, expression values are represented as colors, where the range of colors shows the range of expression values (high, moderate, low, lowest). **F-G** Enrichment of the indicated ranked comparison (1x_MTX vs pre_MTX; 4x_MTX vs 1x_MTX and 4x_MTX vs pre_MTX) monocyte transcriptomes using the genes significantly modulated by AhR in macrophages [28] and antifolates MTX or PMX in GM-CSF-primed macrophages (GM-MØ) for the complete cohort (RA & PsA patients) (**F**) and the independent arthritis groups (PsA, left) and RA (right) (**G**)**.** The intensity of color increases with the enrichment of the gene signature. FDRq value is defined by the size of the corresponding circle

Based on the above results, and considering that monocytes and monocyte-derived macrophages play a key pathogenic role in inflammatory arthritis [14, 16], we undertook the analysis of the kinetics of MTX in monocytes from twenty-nine DMARD therapy-naïve patients with early arthritis, 22 patients with RA and 7 patients with peripheral polyarticular PsA (METOMAC-PAC observational study) whose demographics, baseline characteristics and clinical response [Partial (PR) or Good (GR)] are shown in Tables 1 and 2. Monocytes were isolated at baseline (pre-MTX), and 5 days after the first MTX intake (1 × MTX, in accordance to results from METOMAC pilot study, one-dose) and fourth MTX dose (4 × MTX), and MTX-polyglutamylation (MTX-PG) and RNAseq was determined (Fig. 2A-B, Suppl. Figure 3). Importantly, although MTX-PG accumulation increased in the CD14neg fraction between the first and fourth dose (Suppl. Figure 3), total MTX-PGs were very significantly higher (tenfold) in CD14pos than in CD14neg cells and RBC [13] (Fig. 2C), suggesting monocytes as key MTX targets. Indeed, MTX-PG was detected in monocytes from all patients after the start of the treatment, albeit with a large inter-individual variation (Fig. 2D). The MTX-PG distribution profile was mainly composed of MTX-PG1 (60%) and to a lesser extent of MTX-PG2 (20%), MTX-PG3 (10%) and MTX-PG4-5 (5%) (Fig. 2E). Of note, MTX-PG distribution and levels in monocytes did not change one month after the start of the therapy (Fig. 2E). Stratification by diagnosis showed a 30% lower total MTX-PG accumulation in monocytes from PsA vs RA patients in the 4xMTX condition, whereas no differences were found between Good (GR) and Partial responders (PR) (Fig. 2F-G). As human monocytes half-life in the vascular compartment is around one (classical monocytes) to seven days (non-classical monocytes) [25], the presence of MTX-PG in monocytes 5 days after the start of the treatment suggest that MTX polyglutamylation might take place in myeloid precursor cells in the bone marrow [26]. Interestingly, the FPGS enzymatic activity was significantly lower (~ twofold, p < 0.001) in CD14pos than in CD14neg cells (Fig. 3H), whereas RBC lacked FPGS activity (not shown). As a whole, these results indicate that CD14 + monocytes are the major cellular reservoirs of MTX-PG in blood from arthritis patients, with the kinetics of MTX-PG accumulation suggesting drug uptake by bone-marrow monocyte precursors.

**Table 1:** Baseline characteristics of patients enrolled in the METOMAC-PAC study

		RA (n=22)	PsA (n=7)	Total (n=29)
Sex*	Male	4 (18.2)	5 (71.4)	9 (31.0)
	Female	18 (81.8)	2 (28.6)	20 (69.0)
Age (y)**		62.7 (51.1-72.6)	34.6 (32.6-47.0)	58.8 (46.0-69.1)
Ethnicity	Caucasian	14 (63.6)	6 (85.7)	20 (69.0)
	Hispanic	8 (36.4)	1 (14.3)	9 (31.0)
Smoking habit	Never	13 (59.1)	5 (71.4)	18 (62.1)
	Ex-smoker	7 (31.8)	2 (28.6)	9 (31.0)
	Active smoker	2 (9.1)	0 (0)	2 (6.9)
Coffee^1^	No	6 (28.6)	4 (57.1)	10 (35.7)
	Yes	15 (71.4)	3 (42.9)	18 (64.3)
RheumatoidFactor*	No	10 (45.5)	7 (100)	17 (58.6)
	Yes	12 (54.5)	0 (0)	12 (41.4)
ACPA**	No	9 (40.9)	7 (100)	16 (55.2)
	Yes	13 (59.1)	0 (0)	13 (44.8)
MTX dose (mg/wk)		12.5 (10.0-15.0)	12.5 (12.5-15.0)	12.5 (11.3-15.0)
Previousprednisone*	No	11 (50.0)	7 (100)	18 (62.1)
	Yes	11 (50.0)	0 (0)	11 (37.9)
HAQ^4^		1.2 (1.0-1.7)	0.6 (0.4-0.9)	1.0 (0.6-1.6)
CRP (mg/L)^1^		8.0 (4.0-16.8)	7.9 (4.5-15.2)	7.9 (4.0-16.4)
DAS28-ESR^2^		4.7 (4.1-5.7)	4.4 (3.6-4.8)	4.5 (3.9-5.4)
Clinical response	Partial	11 (50.0)	5 (71.4)	16 (55.2)
(3mo post-MTX)	Good	11 (50.0)	2 (28.6)	13 (44.8)

Quantitative data are presented as median (IQR). Qualitative data are presented as count (percentage of subgroup). ^n^Data of n patients were missing

RA, rheumatoid arthritis; PsA, psoriatic arthritis; ACPA, anti-citrullinated protein antibodies; ESR, erythrocyte sedimentation rate; CRP, C reactive protein; DAS28, Disease Activity Score in 28 joints; HAQ, Health Assessment Questionnaire

*p<0.05; **p<0.01; ***p<0.001

**Table 2:** Treatment and disease activity during the METOMAC-PAC study in partial responders (PR) and good responders (GR) group

			Baseline			3 months	
		PR (n=16)	GR (n=13)	Total (n=29)	PR (n=15)	GR (n=12)	Total (n=27)
MTX dose (mg) ^$^		12.5 (12.5-15.0)	12.5 (10.0-15.0)	12.5 (11.3-15.0)	15.0 (15.0-17.5)^^	12.5 (12.5-15.0)*	15.0 (12.5-15.0)^^
MTX route	Oral	15 (93.8)	12 (92.3)	27 (93.1)	9 (60.0)^	11 (91.7)	20 (74.1)
	ubcutaneous	1 (6.2)	1 (7.7)	2 (6.9)	6 (40.0)	1 (8.3)	7 (25.9)
Prednisone	Yes	9 (56.3)	9 (69.2)	18 (62.1)	8 (53.3)	6 (50.0)	14 (51.9)
	No	7 (43.8)	4 (30.8)	11 (37.9)	7 (46.7)	6 (50.0)	13 (48.1)
Prednisone		7.5 (5.0-12.5)	10.0 (10.0-15.0)	10.0 (5.6-13.8)	5.0 (5.0-7.5)	5.0 (4.4-6.3)	5.0 (5.0-6.3)^
Patient Global VAS		5.0 (3.5-7.0)	4.0 (3.1-6.0)	5.0 (3.3-6.9)^1^	3.5 (2.0-8.0)	2.0 (1.5-3.5)^	3.0 (1.9-4.3)^⁵
Patient Pain VAS		6.0 (4.5-7.1)	3.0 (2.4-7.1)	4.9 (3.0-7.1)^3^	5.0 (2.4-7.0)	2.0 (0.8-2.9)*^^	2.8 (1.6-5.5)^^¹¹
Tender joint count (28)		6 (3-9)	4 (2-7)	6 (2-8)	3 (0-7)^	1 (0-1)^^	1 (0-4)^^^⁵
Swollen joint count (28)		4 (3-6)	5 (4-8)	4 (4-6)	2 (1-5)	1 (0-1)*^^	2 (0-2)^^⁴
Physician Global VAS		4.9 (4.0-6.0)	4.5 (3.0-6.0)	4.8 (3.8-6,0)^2^	3.0 (2.0-5.0)	1.4 (1.0-2.0)***^^	2.0 (1.0-3.1)^^⁵
HAQ		1.1 (0.6-1.7)	1.0 (0.6-1.6)	1.0 (0.6-1.6) ^4^	1.5 (0.8-2.1)^	1.0 (0.3-1.4)	1.3 (0.6-1.9) ¹⁴
DAS28-ESR		4.6 (4.0-5.7)	4.5 (3.8-5.3)	4.6 (4.0-5.4)^2^	3.6 (2.4-5.1)^	2.8 (2.3-3.5)^^	3.2 (2.5-4.4)^^^ ⁹
CRP (mg/L)		8.0 (5.5-16.8)	6.7 (3.5-17.0)	8.0 (4.0-16.4)^1^	8.0 (4.3-15.1)	4.0 (1.4-4.4)*^	4.6 (4.0-9.4)^⁵

Quantitative data are presented as median (IQR). Qualitative data are presented as count (percentage of subgroup)

VAS, visual analogue scale; ESR, erythrocyte sedimentation rate; CRP, C reactive protein; DAS28, Disease Activity Score in 28 joints; HAQ, Health Assessment Questionnaire

^n^Data of n patients were missing

^$^For basal visit values correspond to MTX dose prescribed, basal MTX dose was 0 before study beginning

*p<0.05; **p<0.01; ***p<0.001 for GR vs PR comparison. ^p<0.05; ^^p<0.01; ^^^p<0.001 for basal vs 3months visits comparison.

### METOMAC-PAC observational study: the transcriptional effect of MTX

We have previously demonstrated that MTX treatment causes a huge transcriptional shift in monocyte-derived macrophages [17]. Thus, given the high levels of MTX-PG in monocytes from arthritis patients treated with MTX, mRNA sequencing was performed on CD14^pos^ monocytes before (pre-MTX), and after (1 × MTX, 4 × MTX) (Fig. 3A). Application of the partial least squares-discriminant analysis (PLS-DA) model showed that the transcriptomes of pre-MTX and 1xMTX monocytes are similar, and revealed significant differences between the transcriptomes of 4xMTX and pre-MTX monocytes, as well as between 4 and 1xMTX monocytes (Fig. 3B) indicating that, *in vivo*, MTX modulates the transcriptional signature in monocytes only after 4 shots. Specifically, 233 differentially expressed genes (*p* < 0.05) were found when comparing the transcriptome of 4xMTX and pre-MTX monocytes, with 4xMTX monocytes exhibiting significantly diminished expression of 27 genes, including *ADORA1* (Fig. 3C), and augmented levels of 206 genes, including those associated to chemotaxis function like *CCL2*, *CSF1* and *CCL7* (Fig. 3C-D). Of note, Gene Set Enrichment Analysis (GSEA) revealed that 4 × MTX monocytes show an enrichment of genes upregulated by MTX as well as a significantly reduced expression of the genes inhibited by MTX in monocyte-derived macrophages *in vitro* (GSE71253) [17] (Fig. 3E, Suppl. Table 2). Indeed, leading edge analysis evidenced that 4 × MTX monocytes exhibit an upregulation of numerous genes that characterize the macrophage MTX-response, including *TNFAIP3, LIF, IL1B* and *GDF15* (Fig. 3E), and similar results were obtained with multi-targeted anti-folate pemetrexed (PMX)-dependent genes in macrophages (GSE159349) [27] (Fig. 3E). Therefore, sustained exposure to MTX (4 shots) is required for MTX to modulate the transcriptome of monocytes from RA patients *in vivo* towards the acquisition of MTX-regulated genes as well as to upregulate the expression of genes directly to chemotactic functions.

Further GSEA revealed an additional effect of MTX, as the transcriptome of 4xMTX monocytes exhibited a very significant enrichment of the genes that define the aryl-hydrocarbon receptor (AhR) response [28], a xenobiotic receptor that activates a pleiotropic transcriptional response after binding ligands containing aryl hydrocarbon rings (Fig. 3F). This finding is specially significant because expression of both *AHR* and *CYP1A1*, the prototypic AHR responsive genes, is significantly lower in PBMCs from RA patients, and because folic acid functions as a competitive antagonist of AhR [29, 30]. In this regard, and like in the case of MTX-regulated genes, the enrichment in AhR-response genes increased along MTX treatment (Fig. 3F). Moreover, the same GSEA profiles were obtained stratifying by diagnosis (Fig. 3G). Considering that AhR limits inflammation [31] and MTX increases the expression of the *TNFAIP3* gene, which encodes the negative regulator of NFκB signaling A20 protein, itself an RA susceptibility gene [18, 32, 33], these results indicate that sustained exposure to MTX lowers the global inflammatory state of monocytes *in vivo*.

### The transcriptional effect of MTX in monocytes from good responder and partial reponder patients

**Fig. 4:**
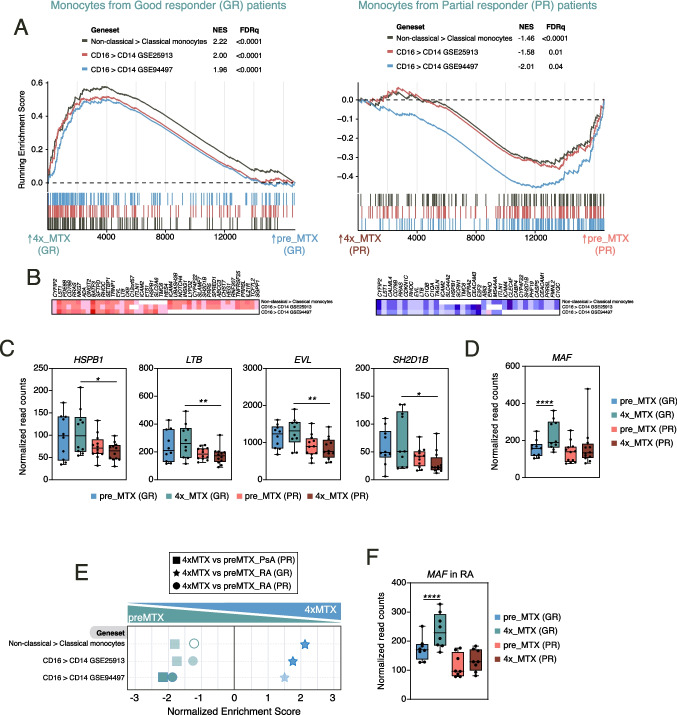
The transcriptional effect of MTX in monocytes from Good and Partial Responder patients. **A** GSEA on the ranked comparison of the 4x_MTX versus pre_MTX monocyte transcriptomes in good responders (GR) (left) and partial responders (PR) (right) patients (complete cohort, RA & PsA), using the genes significantly overexpressed in non-classical (CD16^ +^) relative to classical (CD14^ +^) monocytes (GSE25913, GSE94497) as data set. Normalized Enrichment Score (NES) and False Discovery Rate (FDRq) are indicated. **B** Leading edge analysis of the GSEA of the genes that define CD16 ^+^ monocyte subset on the ranked comparison of the transcriptomes of 4x-MTX versus pre-MTX groups. In the heatmap, expression values are represented as colors, where the range of colors shows the range of expression values (high, moderate, low, lowest). **C-D** Relative expression of the indicated genes as determined by RNA-sequencing on monocytes on the indicated timepoint (pre-MTX and 4xMTX) in good responder (GR) and partial responders (PR) patients. Mean ± SEM of 10–12 patients are shown (*p < 0.05, **p < 0.01, ****p < 0.0001, DESeq2 analysis). **E** GSEA on the ranked comparison of the 4x_MTX versus pre_MTX monocyte transcriptomes in good responders (GR) for RA patients and partial responders (PR) for RA or PsA patients, using the genes significantly overexpressed in non-classical (CD16^ +^) relative to classical (CD14.^ +^) monocytes (GSE25913, GSE94497) as data set. Normalized Enrichment Score (NES) and False Discovery Rate (FDRq) are indicated (FDR > 0.05 empty symbol, FDR < 0.05 transparency of symbol, FDR < 0.001 full symbol color). **F** Relative expression of *MAF* as determined by RNA-sequencing on monocytes on the indicated timepoint (pre-MTX and 4xMTX) in good responder (GR) and partial responders (PR) RA patients. Mean ± SEM of 8 RA patients are shown (****p < 0.0001, DESeq2 analysis)

We next examined changes in the monocyte transcriptome of inflammatory arthritis patients according to their clinical response to MTX (Table 2). We had previously demonstrated that the concentration of soluble CD14 (sCD14) in peripheral blood plasma and serum decreases only in MTX-responder RA patients [27]. Since sCD14 levels reflect the expression of membrane-bound CD14 (mCD14) [34], whose presence defines the three major human monocyte subsets (classical: CD14^++^/CD16^−^; intermediate; CD14^+^/CD16^+^; non-classical: CD14^dim^/CD16^+^) [25], we hypothesized that the relative levels of the three monocyte subsets might be differentially altered by MTX in Good (GR) and Partial responder (PR) patients. To that end, we analyzed the expression of the genes that define classical, intermediate and non-classical monocytes in the transcriptome of both GR and PR patients after four shots of MTX (4xMTX). The transcriptome of monocytes from GR patients showed a robust positive enrichment in the geneset that defines non-classical monocytes, as described by the FANTOM Consortium (Fig. 4A) [35]. Furthermore, identical results were observed upon analysis of non-classical monocyte-specific genes identified in two additional independent studies (GSE25913, GSE94497) [36, 37]. Of note, an opposite effect was seen in the case of PR patients monocytes, whose transcriptome showed a significant decrease in the expression of the genesets that define non-classical monocytes (Fig. 4A). Therefore, the genes that mark the non-classical human monocyte subset are specifically increased in the transcriptome of the monocytes of patients with a good clinical response to MTX (GR), while the opposite occurs in ther case of partial responders (PR). This finding suggests that augmented relative levels of non-classical monocytes reflect, and might predict, a good clinical response to MTX, a prediction that supports and extends our previous findings on sCD14 as a biomarker for MTX response [27]. In fact, leading egde analysis identified the best non-classical monocyte-specific genes that predict MTX response (*CYFIP2*, *LST1*, *CD79B*, *RRAS*, *RHOC*, *EVL*, *LTB*, *ITLN1*, *ICAM2*, *HSPB1*, *TMC6*, *ICAM4*, *CYP4F22*, *SH2D1B*, *PRR5L*) (Fig. 4B). Indeed, the expression of four of these genes (*HSPB1*, *LTB*, *EVL* and *SH2D1B*) was found to be markedly different between GR and PR patients monocytes after 1 month of MTX treatment (Fig. 4C). Further supporting these results, the transcriptome of GR and PR patients monocytes after 1 month MTX treatment also differed in the expression of the CD16 + monocyte-containing cluster #1 defined in the MoMac-VERSE, a resource that identifies conserved monocyte and macrophage states and global imprinting across human tissues (Suppl. Figure 4) [38]. Furthermore, comparison of the gene profiles of 4xMTX versus pre-MTX in monocytes from GR and PR revealed that the gene expression levels of *MAF* was only upregulated in GR patients monocytes (Fig. 4D), a trait that was also significant after adjusting for gender and MTX dose (Suppl. Figure 5, ΔMAF). The exclusive upregulation of *MAF* in GR monocytes after MTX treatment is of particular relevance because MAF marks non-classical monocytes [36, 37], limits macrophage activation [39], regulates reparative genes in macrophages [40], and interacts with AhR [41], whose target genes are specifically regulated by MTX (see Fig. 3F). Finally, similar results were obtained analyzing independently the PsA group for partial responders patients (Fig. 4E) and the RA group for partial and good responders patients (Fig. 4E-F).

### The transcriptional profile of non-classical monocytes is also enriched in monocytes from good responder patients before MTX treatment

**Fig. 5:**
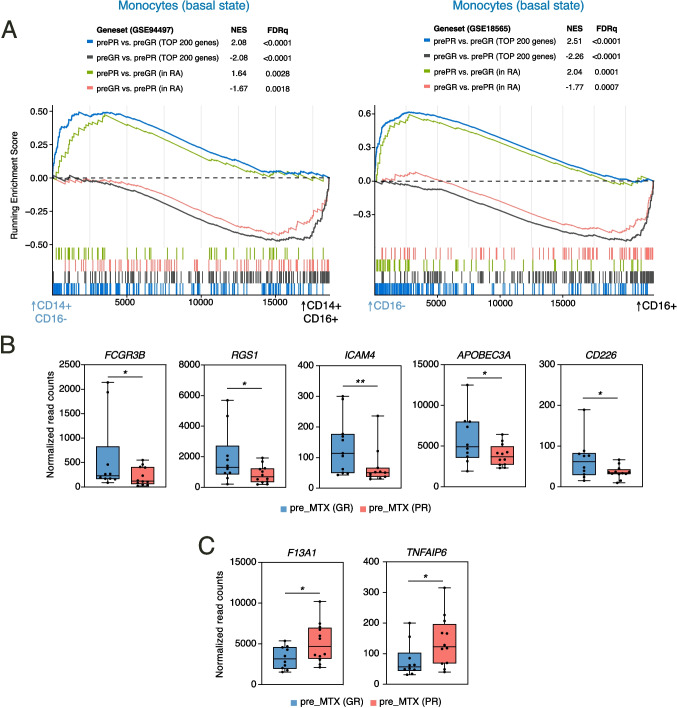
Baseline monocyte transcriptional profile from Good and Partial Responder patients. **A** GSEA on the ranked comparison of classical (CD14 +  + CD16 -) versus non-classical (CD14 + CD16 +) monocyte transcriptomes (GSE94497, left and GSE18565, right) using the genes significantly overexpressed in good responders (GR) and partial responders (PR) patients from the complete early arthritis cohort (RA & PsA) or the genes significantly overexpressed in RA good responders (GR) and RA partial responders (PR) patients, as data set. Normalized Enrichment Score (NES) and False Discovery Rate (FDRq) are indicated. **B-C** Relative expression of the indicated genes as determined by RNA-sequencing on monocytes before MTX treatment (pre_MTX) in GR and PR patients. Mean ± SEM of 10–12 patients are shown (*p < 0.05, **p < 0.01, DESeq2 analysis)

Given the above findings, we assessed the expression of non-classical monocyte-specific genes in GR and PR patients monocytes before MTX treatment (pre-MTX). GSEA on the ranked comparison of the transcriptomes of classical and non-classical monocyte subsets from two independent studies [42, 43] showed that pre-MTX monocytes from GR patients expressed higher levels of genes associated to non-classical monocytes (Fig. 5A). Conversely, pre-MTX monocytes from PR patients expressed higher levels of genes associated to classical monocytes (Fig. 5A). Comparable results were found for pre-MTX monocytes exclusively in good and partial responder RA patients (Fig. 5A). These results imply that the clinical response to MTX might be determined by the baseline transcriptome of CD14 + monocytes, where atypically higher expression of non-classical monocyte-specific genes associates to a better clinical response to MTX. In fact, the expression of non-classical monocyte-specific genes like *FCGR3B* (encoding CD16), *ICAM4*, *APOBEC3A* and *CD226* was significantly higher in pre-MTX monocytes from GR patients than PR patients (Fig. 5B), while expression of classical monocyte-specific genes like *F13A1* and *TNFAIP6* showed an opposite trend (Fig. 5C). Indeed, these differences remained significant after adjustement by gender and MTX dose (Suppl. Figure 5). Therefore, baseline expression of *FCGR3B, ICAM4, APOBEC3A* and *CD226* appear as robust predictors for clinical response to MTX treatment.

### Machine learning using support vector machines (SVM) and random forest (RF) could predict the response to MTX treatment based on the expression of selected genes

**Fig. 6:**
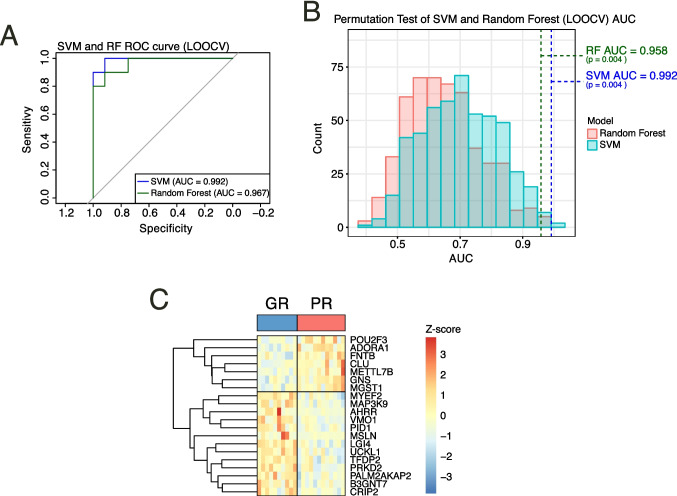
Machine learning models on the response to MTX treatment based on the expression of selected genes. **A** ROC curves Support Vector Machine (SVM, blue line) and Random Forest (RF, green line) classifiers trained on the top 20 genes (ranked by p-value) plus covariates from monocytes before MTX treatment. **B** Permutation tests (500 iterations) for SVM and RF models, the observed AUC is indicated by a blue (SVM) or green (RF) dotted line. **C** Heatmap visualization of the top 20 genes used in the trained models, scaled by z-score, in the patients classified as Good responders (GR) or Partial responders (PR) from the complete early arthritis cohort (RA & PsA)

To evaluate whether the monocyte transcriptome can serve as a predictor of response to MTX treatment, we performed a machine learning model using the top 20 genes ranked by *p-value* from the DESeq2 analysis in pre-MTX condition, incorporating sex, ethnicity, and seropositivity as covariates. Both SVM and RF models, evaluated with leave-one-out cross-validation (LOOCV), achieved excellent accuracy in distinguishing good responders from partial responders to MTX treatment (Fig. 6A). To ensure that predictive performance was not attributable to random variation, a permutation test was conducted in which treatment responses were randomly reassigned. The resulting permutation *p-value* (*p* = 0.004) for both analysis confirmed that the predictive performance of the SVM and RF model was driven by the top 20 gene expression patterns and co-variates rather than chance (Fig. 6B). A heatmap visualization further illustrated the expression patterns across patients, revealing clustering according to up- or downregulation of genes and response category to MTX treatment (Fig. 6C). Although the SVM and RF models demonstrated high apparent predictive performance, these results should be interpreted with caution given methodological constraints, most notably the small sample size, and the current findings should be considered as hypothesis-generating. Altogether, these results indicate that monocyte transcriptomic signatures could have strong potential as predictors of MTX treatment response.

## Discussion

In the present observational study we have evaluated MTX intracellular retention in blood cells from early arthritis patients that begin low-dose MTX therapy, and later assessed the influence of MTX-PG levels on the molecular inflammatory state of monocytes. The METOMAC-PAC study revealed that MTX-PG accumulates specifically in peripheral blood monocytes, where MTX-PG are detected 5 days post-treatment, suggesting MTX-PG accumulation by myeloid precursors. Transcriptional analysis of monocytes revealed that MTX modulates the monocyte gene profile only after 4 MTX doses, with specific enrichment of the aryl-hydrocarbon receptor (AhR)-molecular signature and augmented expression of genes coding for anti-inflammatory factors (*CCL2*, *M-CSF*). Importantly, stratification of METOMAC-PAC patients according to clinical response (Good Responders, GR; Partial Responders, PR) revealed that GR patients exhibit increased expression of “non-classical monocyte”-specific genes (*MAF*, *FCGR3B*, *ICAM4*) both before and after MTX treatment, while PR patients showed a higher baseline expression of genes preferentially expressed by “classical monocytes”.

Novel mass spectrometry-based procedures for the analytical determination of MTX-PG in low cell numbers has allowed us to demonstrate, for the first time, the presence of MTX-PG in blood monocytes as early as five days after initiation MTX administration, what gives important clues about the pharmacokinetics of MTX and its cellular targets. Specifically, and considering the lifespan of the three peripheral blood monocyte (PBM) subsets (one, four and seven days for classical, intermediate and non-classical monocytes, respectively), and the accelerated monocytopoiesis observed in RA patients [25, 44], our findings indicate that initial formation of MTX-PG takes place in myeloid precursor cells in bone marrow because up to 85% of PBM will be newly formed at day 5 after the first MTX shot (Suppl. Figure 6). Supporting this suggestion, MTX has been shown to accumulate in bone marrow cells *in vitro* and also *in vivo* in lymphoma patients after treatment with high-dose MTX [26, 45]. The folylpolyglutamate synthetase (FPGS)-dependent formation of MTX-PG in bone marrow myeloid precursors also provides a reasonable explanation to the fact that MTX-PG accumulation is higher in monocytes than in lymphocytes, in spite of their lower FPGS activity, because FPGS catalytic activity and folate metabolism is higher in cells with high proliferative capacity (e.g., myeloid precursor cells) than in resting or terminally differentiated cells (e.g., as monocytes) [46].

Temporal transcriptional analysis of monocytes indicates that MTX modulates the monocyte transcriptional signature 26 days after the start of treatment. In agreement with our previous finding on the MTX training-tolerance-like effect on macrophages *in vitro* [17], the METOMAC-PAC study here reported reveals a similar change in gene profile *in vivo*, suggesting that MTX can access the bone marrow and reprograms myeloid precursors to generate monocytes with a modified ("tolerized") metabolic and transcriptional profile, and thus with reduced capability for inflammatory cytokine production upon exposure to danger signals present in the inflammed synovia. Along this line, the transcriptome of 4xMTX monocytes *in vivo* also exhibit a very significant over-representation of the genes that define aryl-hydrocarbon receptor (AhR) response. The relevance of this finding is illustrated by the anti-inflammatory consequences of AhR activation [31] and its involvement in endotoxin tolerance [47]. Besides, PBMCs from healthy individuals exhibit higher expression of *AHR* and the AhR-dependent gene *CYP1A1* than RA patients, thus suggesting the MTX capacity to restore homeostatic AhR levels and/or activity in blood monocytes [30]. In the context of antifolates like MTX, its link to the AhR pathway is of particular relevance because reduced folate carrier (RFC) and the ABC drug transporter ABCG2, the transporters that mediate the cellular influx/efflux of MTX (Suppl. Figure 7), are AhR targets [48–50] and because folic acid is a natural antagonist of AhR [29]. The enrichment of AhR responssive genes after four MTX doses might reflect a potential action of MTX on the DNA binding ability and/or the transcriptional activity of AhR, both of which have been demonstrated to be impaired by folic acid [29]. Considering that the p-aminobenzoic acid (PABA) is the aryl hydrocarbon portion of folic acid with high binding affinity to AhR, docking analysis could accurately predict whether MTX, with a methyl group on the 10th nitrogen of the PABA ring, binds to AhR. In fact, if MTX binds to AhR, MTX could block the inhibitory actions of folic acid, what would end up allowing the subsequent binding of endogenous ligands to AhR. If this is the case, folic acid supplementation would limit the MTX-triggered increase in AhR-dependent gene signature and, consequently, would impair the MTX-triggered anti-inflammatory effect, an issue that deserves further investigation. Alternatively, the enrichment of AhR responssive genes in 4xMTX patients could be explained by a direct activatory binding of MTX to AhR, although preliminary *in vitro* experiments suggest that this is not the case (not shown).

The results of the METOMAC-PAC study provides novel insights into the molecular processes underlying the diverse patient-specific response to MTX treatment. Discriminating the monocyte transcriptional profile between GR and PR has uncovered that the genes that mark the non-classical human monocyte subset (CD16^+^) are specifically over-repressented in the good responder´s gene profile both before and after MTX treatment. This result is in line with (i) the role of non-classical monocytes in promoting phagocytosis and resolution of inflammation [51, 52], (ii) the lower concentration of plasma sCD14 found only in MTX-responder RA after 6 months of therapy [27] and (iii) the lower expression of CCR2 (that marks classical monocyte subset) in monocytes from MTX-responder patients [53]. Among the “non-classical monocyte”-associated genes identified as predictors for effective MTX clinical response (*FCGR3B*, *ICAM4*, *APOBEC3A* and *CD226*), *CD226* is an RA susceptibility gene in Iranian, Colombian and Chinese Han population, and is associated to anti-TNF response [54, 55], whereas *APOBEC3A* codifies for a cytidine deaminase involved with RNA editing activity in monocytes [56]. Besides, the higher *MAF* expression that we have detected in monocytes from MTX-responsive individuals after one month of MTX monotherapy is particulary relevant, because MAF is a transcription factor involved in IL-10 regulation in macrophages [40], and marks non-classical monocytes [36, 37]. Our findings underscore the potential involvement of non-classical monocytes in mediating MTX response. Given that MTX is the primary treatment for RA and PsA, and considering the critical importance of leveraging the window of opportunity to achieve early remission [57], it would be valuable to monitor the expression of these markers to identify patients most likely to respond to MTX. We recognize that future studies should include a larger validation cohort with flow-cytometry analysis quantifying monocyte subsets and comparing some of the identified genes in isolated populations to strengthen the reliability of these results.

## Supplementary Information

Below is the link to the electronic supplementary material.

Supplementary file1 (PDF 1037 KB)

Supplementary file2 (XLSX 42 KB)

## Data Availability

Data reported in this publication have been deposited in NCBI's Gene Expression Omnibus and are accessible through GEO Series accession numbers GSE279719 and GSE289101.
