# Supplementary material for: Monocytes from inflammatory arthritis patients accumulate methotrexate and their transcriptome predicts methotrexate clinical response
Source: Cell Mol Life Sci. 2026 Mar 17;83(1):178. doi: 10.1007/s00018-026-06162-9 (PMC13013856; doi:10.1007/s00018-026-06162-9)
Supplement: Supplementary file 1 — Supplementary file1 (PDF 1037 KB) [file 18_2026_6162_MOESM1_ESM.pdf]

Supplementary Figures Rios-Schiaffino

S.Table 1. Baseline characteristics of volunteers enrolled in the METOMAC study						
**Volunteer**	**Sex**	**Ethnicity**	**Age (y)**	**Height (m)**	**Weight (kg)**	**BMI**
1	Male	Latin-American	34	1.69	59.8	20.9
2	Male	Caucasian	29	1.77	88.5	28.3
3	Male	Caucasian	23	1.68	63.6	22.5
4	Male	Caucasian	29	1.81	85.3	26.0
5	Male	Caucasian	33	1.79	75.5	23.6
6	Male	Caucasian	21	1.80	84.7	26.1
		**Median**	29.0	1.78	80.1	24.8
		**Minimum value**	21.0	1.68	59.8	20.9
		**Maximum value**	34.0	1.81	88.5	28.3

Supplementary Table 1. Baseline characteristics of volunteers enrolled in the METOMAC study

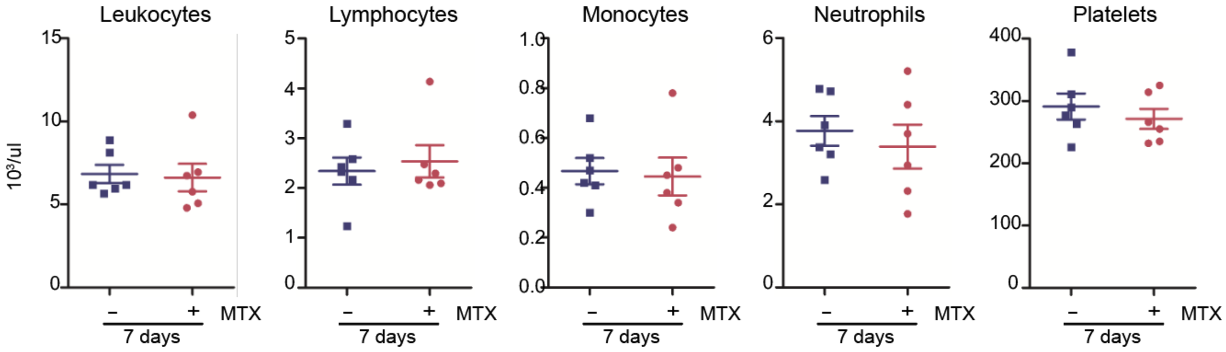

Supplementary Figure 1. METOMAC trail. White blood cell and platelets counts did not differ after MTX intake.

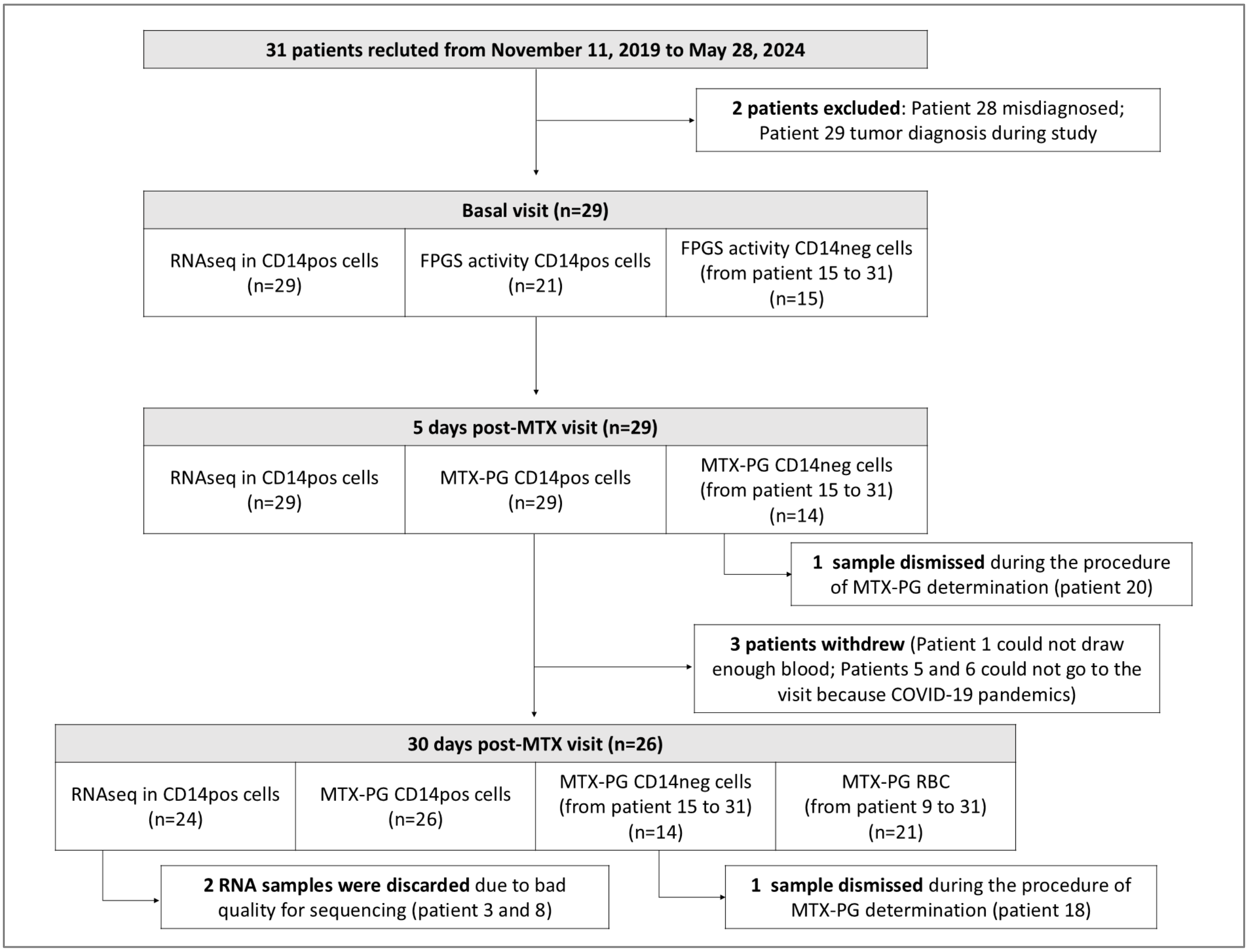

Supplementary Figure 2.- Flow-chart inclusions and exclusions for different molecular determinations in METOMAC-PAC study (RNAseq, MTX-PG, FPGS activity).

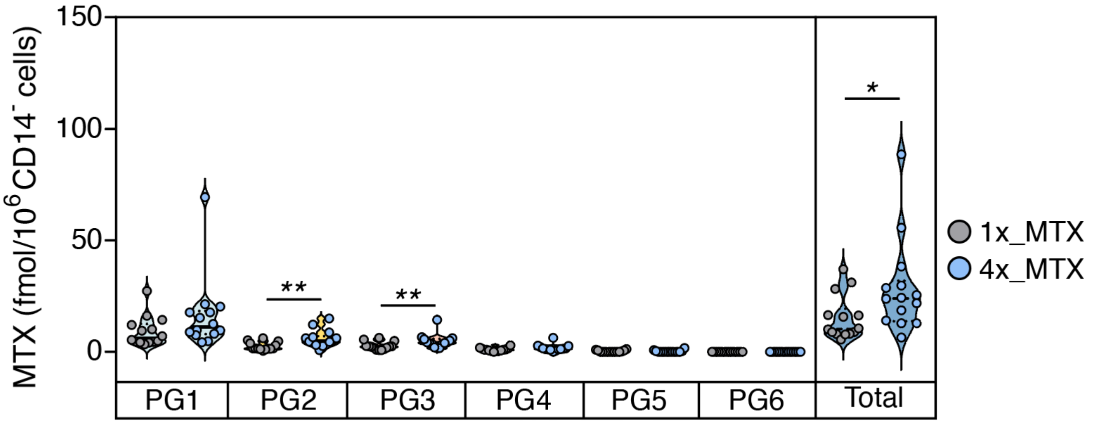

Supplementary Figure 3. MTX-PG concentrations in CD14neg cells in early arthritis patients in 1xMTX (grey, n=14) and 4xMTX (blue, n=14) time points. Individual MTX-PG concentrations (PG1: MTX-PG1; PG2:MTX-PG2; PG3: MTX-PG3; PG4: MTX-PG4; PG5: MTX-PG5; PG6: MTX-PG6) ($p < 0.05$, paired t-test).

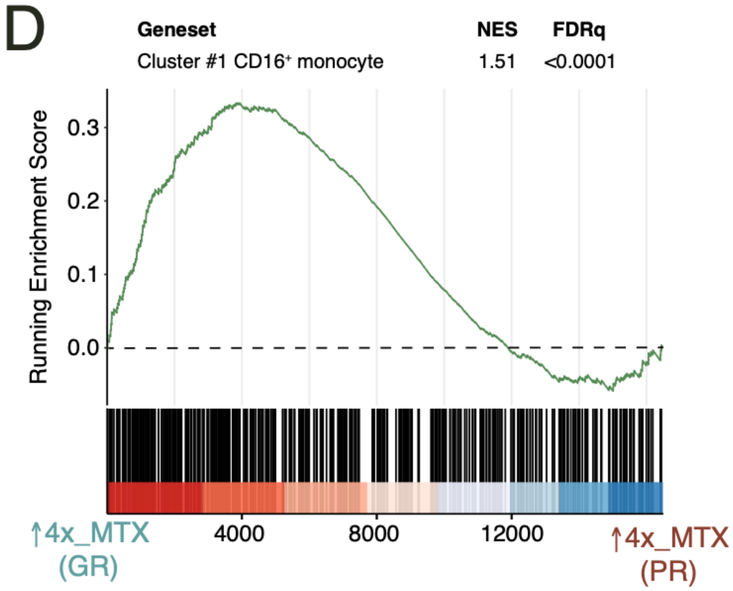

Supplementary Figure 4. GSEA on the ranked comparison of the 4x_MTX monocyte transcriptomes from good responders (GR) and partial responders (PR) patients, using the genes from cluster #1 (CD16+ monocytes) from the MoMac-VERSE, a resource that identifies conserved monocyte and macrophage states and global imprinting across human tissues, as data set. Normalized Enrichment Score (NES) and False Discovery Rate (FDRq) are indicated.

A

	β Coeff. (95% CI)	p-value
**Female gender**	-4.54 (-8.84 to -0.25)	0.03
**MTX dose (mg)**	-0.80 (-1.51 to -0.08)	0.02
**Total MTX-PG (Monocytes)**	-0.0006 (-0.01 to 0.009)	0.89

B

	β Coeff. (95% CI)	p-value
**Female gender**	-4.38 (-8.58 to -0.18)	0.04
**MTX dose (mg)**	-1.20 (-1.95 to -0.45)	0.002
**ΔMAF (read counts)**	0.02 (0.005 to 0.04)	0.01

C

	β Coeff. (95% CI)	p-value
**Female gender**	-4.25 (-7.78 to -0.72)	0.01
**MTX dose (mg)**	-0.62 (-1.14 to -0.106)	0.01
**FCGR3B (read counts)**	0.001 (0.0001 to 0.003)	0.03

D

	β Coeff. (95% CI)	p-value
**Female gender**	-7.10 (-10.78 to -3.42)	< 0.001
**MTX dose (mg)**	-1.52 (-2.37 to -0.67)	< 0.001
**ICAM4 (read counts)**	0.02 (-0.00008 to 0.05)	0.05

E

	β Coeff. (95% CI)	p-value
**Female gender**	-5.69 (- 9.09 to -2.29)	0.001
**MTX dose (mg)**	-1.19 (-2.12 to 0.26)	0.01
**APOBEC3A (read counts)**	0.0006 (0.00006 to 0.001)	0.03

F

	β Coeff. (95% CI)	p-value
**Female gender**	-7.79 (-14.44 to -1.14)	0.02
**MTX dose (mg)**	-1.20 (-2.19 to -0.20)	0.01
**CD226 (read counts)**	0.12 (0.03 to 0.21)	0.008

Supplementary Figure 5. Variables associated with response to MTX (partial response *vs* good response) at 3 months follow-up. Logistic regression multivariable models were developed. Considering the number of patients included in the study, models could only be developed with combination of 3 variables. Female gender and MTX dose were included in all models for better adjustment of the association of those biomarkers discovered in the study. MTX-PG concentration in monocytes **(A)**, ΔMAF expression **(B)**, baseline levels of *FCGR3B* **(C)** baseline levels *ICAM4* **(D)**, baseline levels *APOBEC3A* **(F)**, baseline levels *CD226* **(E)**. ΔMAF= (levels of MAF gene expression after 4X-MTX – levels of MAF gene expression at baseline). Coeff. Coefficient; CI. Confidence interval; MTX. Methotrexate; mg. Milligrams/week.

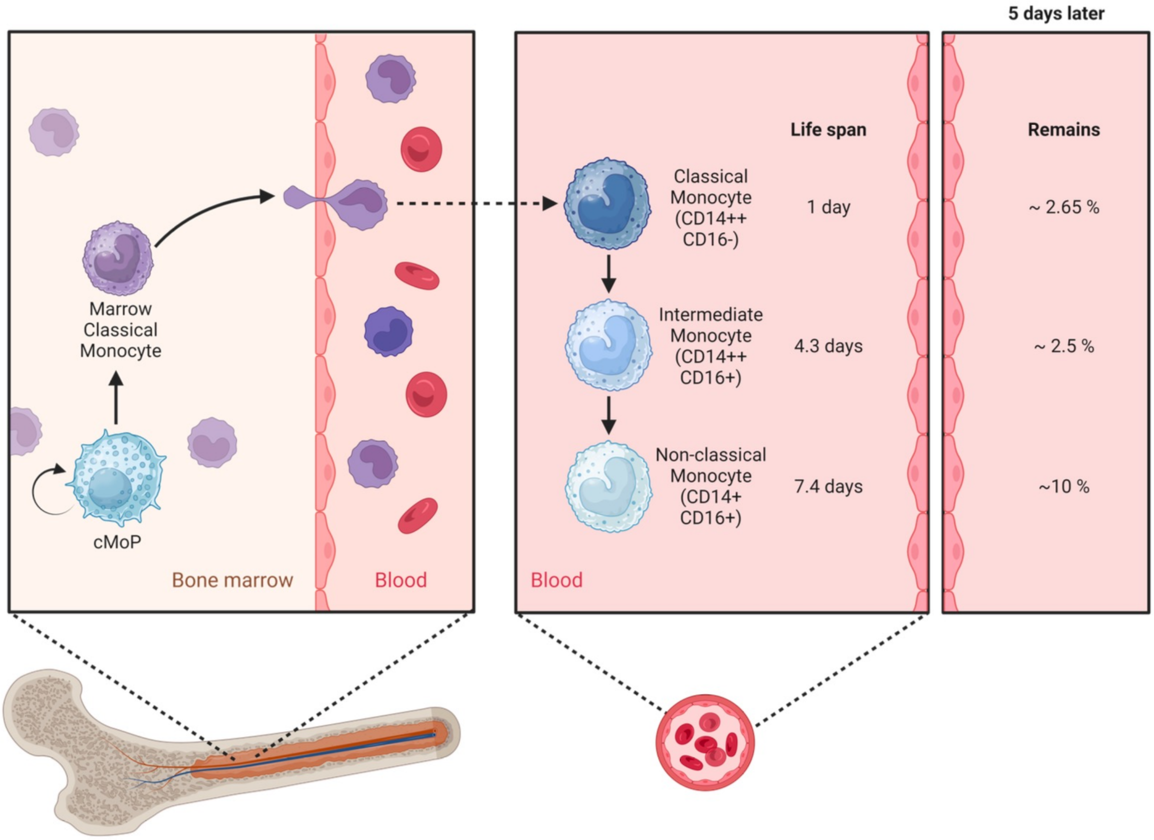

Supplementary Figure 6.- Classical monocytes emerge first from bone marrow after a postmitotic interval of 1.5-2 days and circulate for a day. Classical monocytes contribute around 85-87% to the total monocyte pool, whereas intermediate and nonclassical monocytes make up 5% and 8-10%, respectively. Five days after the first MTX shot around 85% of circulating monocytes will be newly formed.

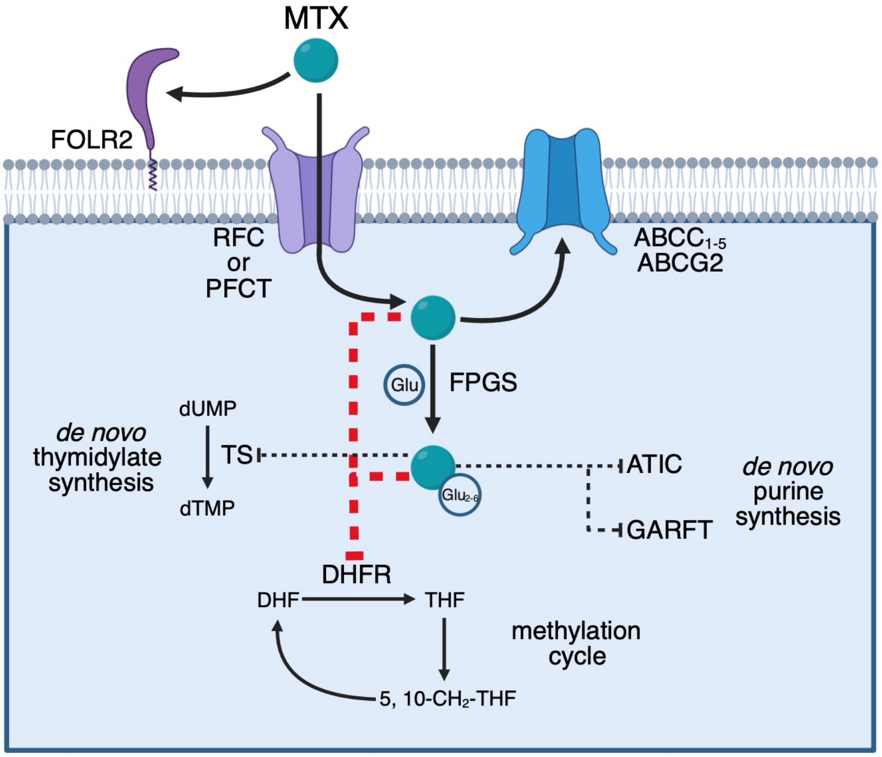

Supplementary Figure 7.- Key components of the MTX metabolism in myeloid cells. MTX enters the cells through reduced folate carrier (RFC), proton-coupled folate transporter (PCFT) and/or folate receptor beta (FRβ), and is substrate for folylpoly-γ glutamate synthetase (FPGS) that catalyzes the addition of glutamate residues. MTX is an inhibitor of DHFR and polyglutamates of MTX also inhibit thymidylate synthase (TS), glycinamide ribonucleotide transferase (GARFT), and aminoimidazole-carboxamide ribonucleotide formyl transferase (ATIC).
